# Does the accuracy of pedicle screw placement differ between the attending surgeon and resident in navigated robotic-assisted minimally invasive spine surgery?

**DOI:** 10.1007/s11701-019-01019-9

**Published:** 2019-09-21

**Authors:** Arnold B. Vardiman, David J. Wallace, Grant A. Booher, Neil R. Crawford, Jessica R. Riggleman, Samantha L. Greeley, Charles G. Ledonio

**Affiliations:** 1 Department of Neurosurgery, University of Texas Health San Antonio, 7703 Floyd Curl Drive, MC 7843, San Antonio, TX 78229-3900 USA; 2 A Division of Globus Medical, Inc, Musculoskeletal Education and Research Center (MERC), 2560 General Armistead Avenue, Audubon, PA 19403 USA

**Keywords:** Robot-navigated, Pedicle screw placement, Minimally invasive, Spine surgery

## Abstract

Robotic assistance with integrated navigation is an area of high interest for improving the accuracy of minimally invasive pedicle screw placement. This study analyzes the accuracy of pedicle screw placement between an attending spine surgeon and a resident by comparing the left and right sides of the first 101 consecutive cases using navigated robotic assistance in a private practice clinical setting. A retrospective, Institutional Review Board-exempt review of the first 106 navigated robot-assisted spine surgery cases was performed. One attending spine surgeon and one resident performed pedicle screw placement consistently on either the left or right side (researchers were blinded). A CT-based Gertzbein and Robbins system (GRS) was used to classify pedicle screw accuracy, with grade A or B considered accurate. There were 630 consecutive lumbosacral pedicle screws placed. Thirty screws (5 patients) were placed without the robot due to surgeon discretion. Of the 600 pedicle screws inserted by navigated robotic guidance (101 patients), only 1.5% (9/600) were repositioned intraoperatively. Based on the GRS CT-based grading of pedicle breach, 98.67% (296/300) of left-side screws were graded A or B, 1.3% (4/300) were graded C, and 0% (0/300) were graded D. For the right-side screws, 97.67% (293/300) were graded A or B, 1.67% (5/300) were graded C, and 0.66% (2/300) were graded D. This study demonstrated a high level of accuracy (based on GRS) with no significant differences between the left- and right-side pedicle screw placements (98.67% vs. 97.67%, respectively) in the clinical use of navigated, robot-assisted surgery.

## Introduction

The traditional teaching method of orthopedic residents for spine surgery is through a preceptorship or apprenticeship approach to orthopedic surgical training with 1:1 training with an attending surgeon, rotating through orthopedic subspecialties. The residents work towards improving comprehensive patient care, along with surgical and clinical skills.

Safe pedicle screw placement may be a factor in achieving positive clinical outcomes as well as avoiding catastrophic complications. Therefore, orthopedic surgical resident education in proper pedicle screw placement is essential. Advances in medical imaging have improved the accuracy of pedicle screw placement, from fluoroscopic-guided to computer-aided navigation. Auode et al. have shown that computer-aided navigation is an effective tool for training orthopedic surgery residents in pedicle screw placement [1]. Free-hand pedicle screw placement includes an increased risk of screw misplacement and a high risk of radiation to the surgeons, operating room staff, and the patients [2]. This has been the motivation for the advancement of navigated robot-assisted spine surgery.

In a recent study analyzing the accuracy of thoracic pedicle screw placement placed by neurosurgery residents, it was found that six postgraduate year (PGY-6) residents were associated with the highest rate of cortex violations compared to PGY-2 and PGY-3 residents [3].

The robot affords a stable platform for guiding pedicle screw placement to pre-planned trajectories, which facilitates accurate pedicle screw placement. Evaluation of pedicle screw accuracy is necessary to determine the efficacy of navigated robotic guidance in spine surgery. To the best of the author's knowledge, pedicle screw accuracy based on the surgeon’s level of training using robotic guidance has not been studied. This study analyzes the accuracy of pedicle screw placement between an attending spine surgeon and a resident by comparing the left and right sides of the first 101 consecutive cases using navigated robotic assistance in a private practice clinical setting.

## Materials and methods

This is a retrospective, Institutional Review Board exempt study of the first 101 navigated robot-assisted spine surgery cases at a single site. Per case, one attending spine surgeon and one resident performed pedicle screw placement consistently on either the left or right side (researchers were blinded). The attending surgeon is a supervising physician in a Committee on Advanced Subspecialty Training accredited spine fellowship, who has been in practice for 24 years. The residents participated in minimally-invasive spinal surgery during their PGY-6 of neurosurgery residency. Radiographic evaluation of preoperative plan and postoperative CT scans was performed and complication rates were collected.

### Navigated robot-assisted pedicle screw positioning system

The robotic positioning system (ExcelsiusGPS^®^; Globus Medical, Inc., Audubon, PA, USA) uses radiological patient images (preoperative CT, intraoperative CT, or fluoroscopy), along with a dynamic reference base and positioning camera to guide pedicle screw placement in real time. Robotic assistance can help guide the surgeon’s planning and approach prior to and during surgery, and is designed to improve pedicle screw accuracy.

### Surgical technique: minimally invasive navigated robot-assisted surgery

In this study, the robotic system operated on one functional modality, intraoperative CT. The image coordinate system was obtained from a portable intraoperative CT (e.g., O-arm, Medtronic SNT, Louisville, CO, USA) or a standard CT scan taken at the time of surgery with the patient already in surgical position (prone). After a CT scan was taken and the spinal levels identified, pedicle screw trajectories were planned and saved. Reference frames were installed and fixated to the pelvis, and instruments and arrays with reflective markers were registered. A surgeon-controlled foot pedal activated and positioned the robot arm to the planned pedicle trajectory. Stab incisions were made on the skin using a scalpel. Pedicle screws were inserted percutaneously using navigated instruments guided by the robotic arm. Per case, one attending spine surgeon and one resident performed pedicle screw placement consistently on either the left or right side. This sequence was repeated until all pedicle screws were placed. Rods were then placed and locking caps were set once the rods were in the proper position. Intraoperative CT images were taken to verify screw and rod position. Interbody devices when used were inserted manually. Surgical incisions were cleaned and closed in the standard fashion.

### Outcome measures

**Fig. 1:**
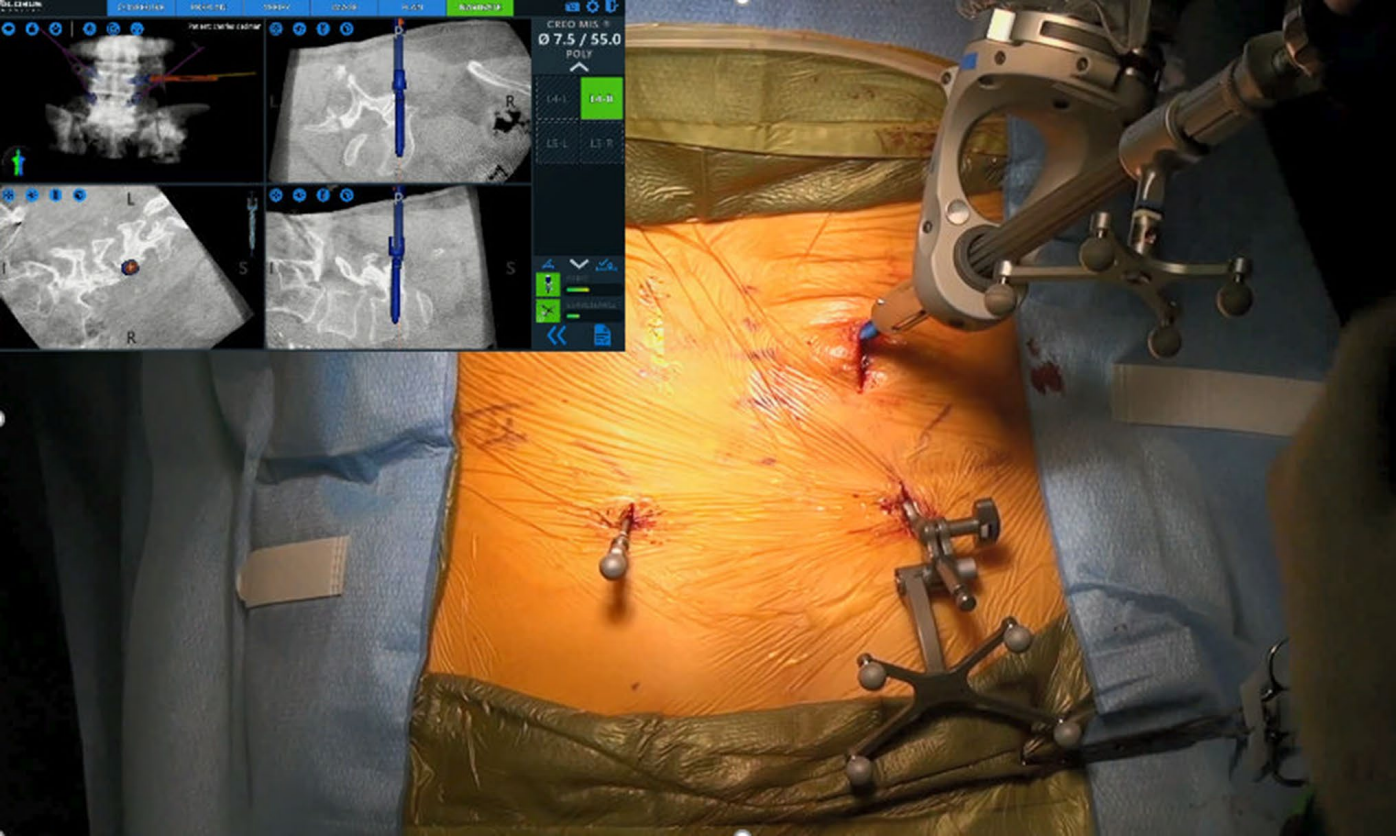
Lumbosacral pedicle screw planning and placement with a minimally invasive navigated robot-assisted pedicle screw positioning system

A CT-based Gertzbein and Robbins System (GRS) was used to classify pedicle screw accuracy, in which screws were graded as A (screw is completely within the pedicle), B (pedicle cortical breach < 2 mm), C (pedicle cortical breach < 4 mm), D (pedicle cortical breach < 6 mm), and E (pedicle cortical breach > 6 mm) [4]. The evaluator was blinded to the study groups. Screws with an A or B grade were deemed as accurate while screws with a C, D, or E grade were considered inaccurate, as previously demonstrated [4–7]. The number of accurate screws divided by the number of total screws placed with robotic navigation resulted in an accuracy percentage for the first 101 cases. Additionally, quantitative three-dimensional screw tip, screw tail, and screw angulation offsets were determined using CT scans and image overlay analysis to compare preoperative planned trajectories to actual postoperative screw placement (Fig. 1). Pedicle screw malposition, reposition, and return to operating room (OR) rates were collected.

### Statistical methods

Statistical analysis was performed using SPSS Statistics Version 25 software (IBM Corp., Armonk, NY, USA). Data were presented as mean ± standard deviation. The level of statistical significance was set to *p* < 0.05 for all statistical analysis.

## Results

### Baseline characteristics

After analysis, it was revealed that pedicle screw placement on the left was performed by the resident and by the attending on right side of the spine. In the first 106 cases, 630 lumbosacral pedicle screws were placed. Thirty screws (5 patients) were placed without the robot due to surgeon discretion. Of the 600 pedicle screws inserted by navigated robotic guidance (101 patients), 300 screws were placed on the left and 300 screws were placed on the right. Only 1.5% (9/600) were repositioned intraoperatively. The average age was 64.8 years, and 55% were female. Average body mass index was 31 kg/m^2^. A majority of the indications were degenerative disc disease (79) and adjacent segment disease (19) (Table 1).

**Table 1:** Baseline characteristics

Parameter	Overall
Number of patients	101
Gender	
Female, *n* (%)	56 (55.4)
Male, *n* (%)	45 (44.6)
Age, mean (SD, range)	64.8 (11.5) (31–87)
BMI, mean (SD, range)	30.6 (5.7) (19–44)
Diagnosis, *n* (%)	
Degenerative disc disease	79 (78.2)
Adjacent segment disease	19 (18.8)
Trauma	2 (2.0)
Infection	1 (1.0)

### Surgical data

Of the 600 screws inserted with navigated robotic guidance, 26.0% (156/600) were performed at L4 and 25.7% (154/600) were performed at L5. The most common screw used was 25% of the time, 7.5 × 50 mm. Average estimated blood loss was 165 cc. Mean operative time was 142 min. The mean length of hospital stay was 4.6 days. The most common disposition was home (32%) and rehabilitation (31%) (Table 2).

**Table 2:** Surgical data

Parameter	Overall
Levels treated, *n* (%)	
L1	16 (2.7)
L2	48 (8.0)
L3	128 (21.3)
L4	156 (26.0)
L5	154 (25.7)
S1	98 (16.3)
Screw size, *n* (%)	
5.5 × 45	2 (0.3)
5.5 × 50	6 (1.0)
5.5 × 55	2 (0.3)
6.5 × 45	9 (1.5)
6.5 × 50	38 (6.3)
6.5 × 55	18 (3.0)
6.5 × 60	11 (1.8)
7.5 × 35	2 (0.3)
7.5 × 40	8 (1.3)
7.5 × 45	56 (9.3)
7.5 × 50	150 (25.0)
7.5 × 55	58 (9.7)
7.5 × 60	20 (3.3)
7.5 × 65	4 (0.7)
8.5 × 40	15 (2.5)
8.5 × 45	48 (8.0)
8.5 × 50	85 (14.2)
8.5 × 55	54 (9.0)
8.5 × 60	12 (2.0)
8.5 × 65	2 (0.3)
Mean estimated blood loss (cc), *n* (SD)	165.4 (92.0)
Mean operative time (min)	142.3 (50.3)
Mean length of hospital stay (days)	4.6 ± 1.9
Disposition, *n* (%)	
Home	32 (31.7)
Rehabilitation	31 (30.7)
Home health	26 (25.7)
Skilled nursing facility	11 (10.9)

### Pedicle screw accuracy

**Fig. 2:**
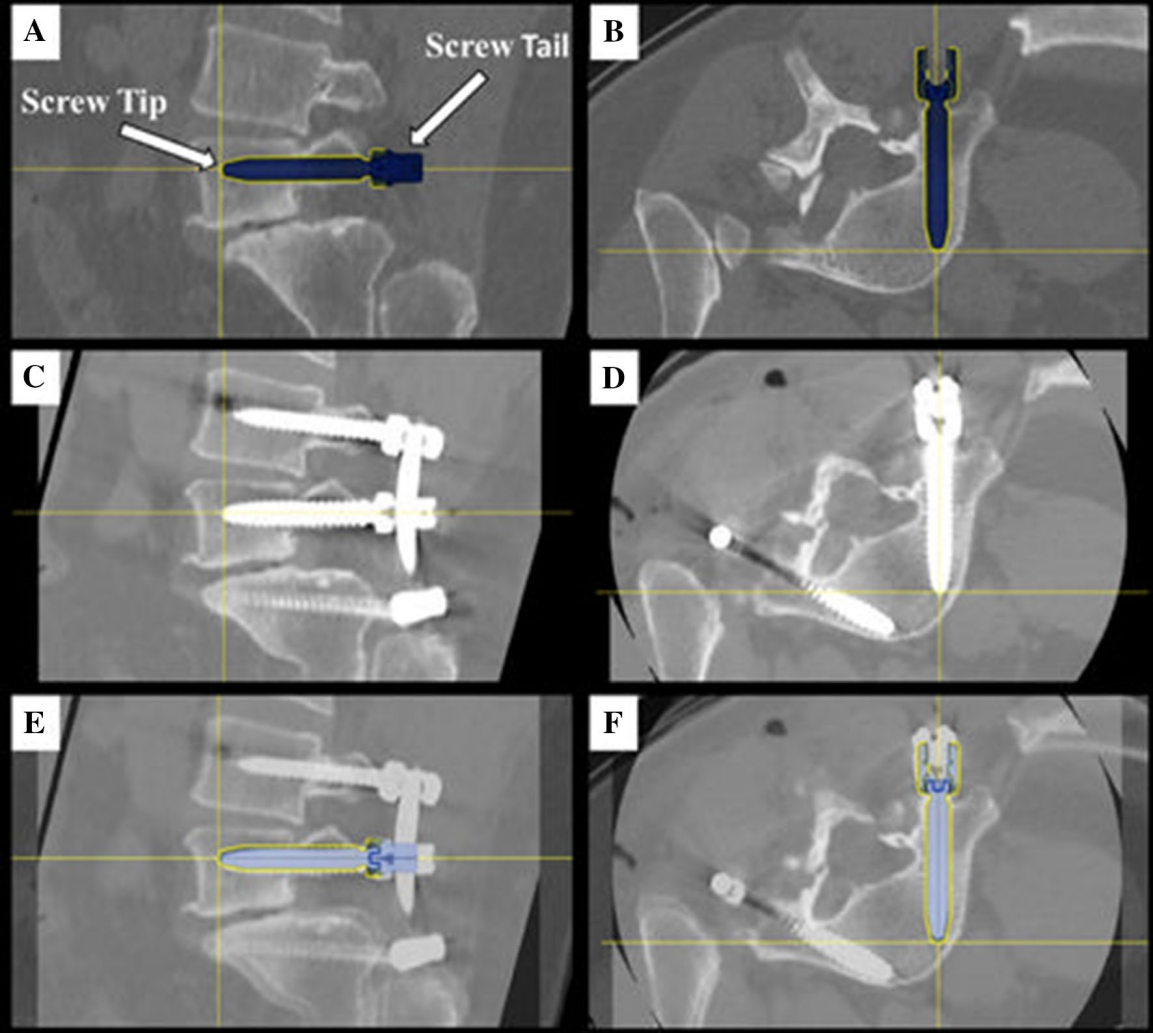
Screw tip, tail, and angle offset assessment. Right L5 screw planning in (**a**) sagittal and (**b**) axial planes. Image overlay analysis with preoperative planned trajectory and postoperative screw placement in (**c**) sagittal and (**d**) axial planes. Postoperative CT of L5 screw placement without a medial or lateral breach in (**e**) sagittal and (**f**) axial planes. The crosshairs indicate screw tip

**Fig. 3:**
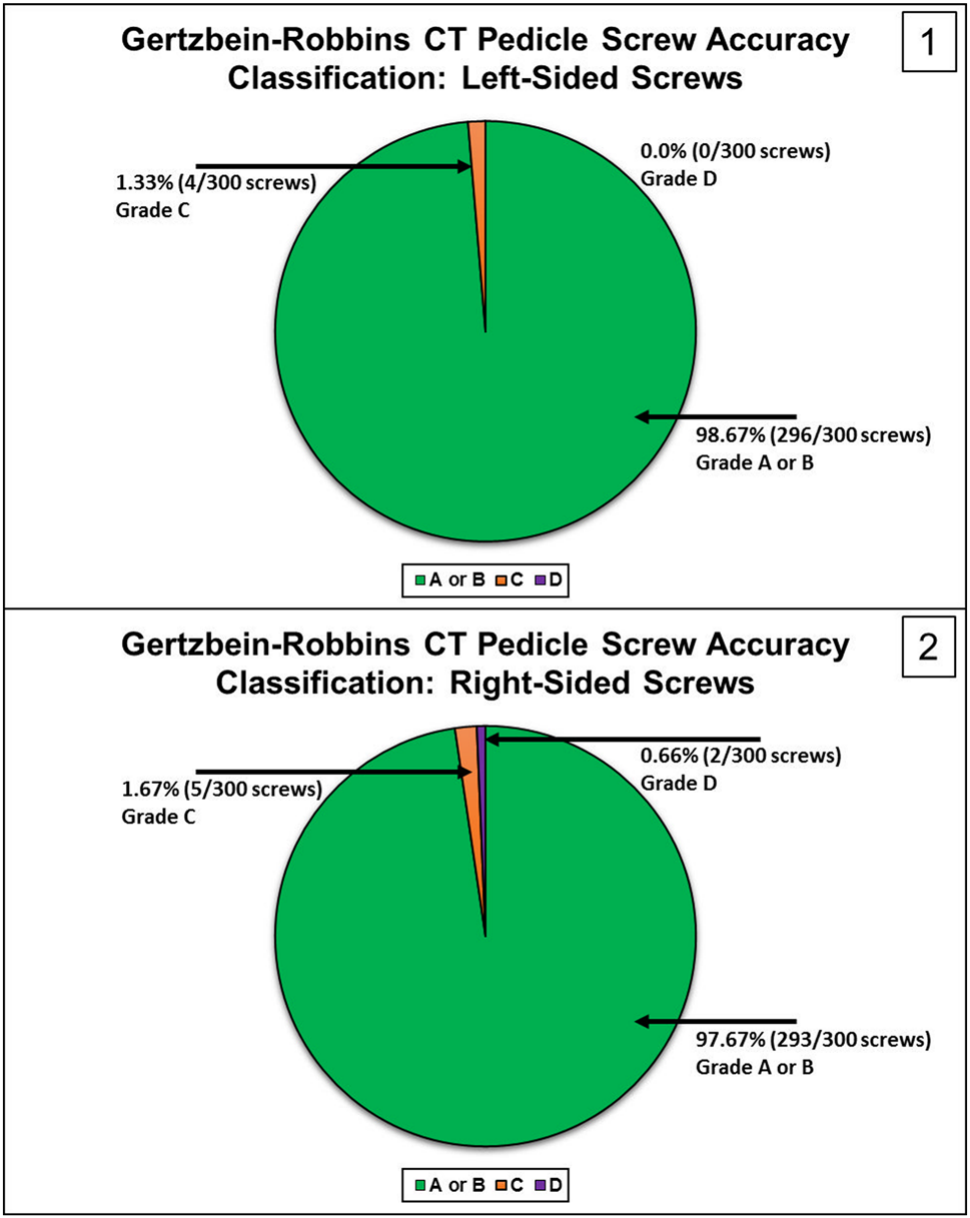
Gertzbein-Robbins CT pedicle screw accuracy classification for left (1) and right-sided (2) screws

Based on the GRS CT-based grading for the left side screws, 98.67% (296/300) were graded A or B, 1.33% (4/300) screws were graded C, and 0% (0/300) screws were graded D (Fig. 2). Based on the GRS CT-based grading for the right-side screws, 97.67% (293/300) were graded A or B, 1.67% (5/300) screws were graded C, and 0.66% (2/300) screws were graded D (Fig. 3). Using a Chi square test, there were no significant differences between the left and right sides in screw accuracy (*p* > 0.05).

The average offset from preoperative plan to actual final placement of left-side (resident) screws was 1.74 ± 1.31 mm from tip, 1.75 ± 1.24 mm from tail, and 1.98 ± 1.43° of angulation. The average offset from preoperative plan to actual final placement of right-side (attending) screws was 1.75 ± 1.39 mm from tip, 1.82 ± 1.14 mm from tail, and 2.11 ± 1.75° of angulation. Using an independent samples *t* test it was found that there were no significant differences between the left and right sides in tip, tail, or angulation offset (*p* > 0.05) (Table 3).

**Table 3:** Translational and angular screw offset

Measurement	Left side	Right side
Tip (mm)	1.7 ± 1.3	1.8 ± 1.4
Tail (mm)	1.8 ± 1.2	1.8 ± 1.1
Angular (°)	2.0 ± 1.4	2.1 ± 1.8

### Complications

Two complications—interbody removal and wound vacuum-assisted closure—were reported as requiring a return to the OR, but these were not related to robotic guidance or pedicle screws.

## Discussion

The accuracy of pedicle screw placement is of the utmost importance for the safety of patients undergoing instrumented spinal fusion. Two- and three-dimensional fluoroscopy along with intraoperative CT for freehand real time navigation based on actual patient anatomy has greatly improved pedicle screw accuracy compared to freehand manual guidance [8]. In a systematic review of 30 studies, Mason et al. reported an overall accuracy rate for the freehand conventional method was 68.1%, for 2-D fluoroscopy navigation was 84.3% and for 3-D navigation was 95.5% [9]. Robotic-assisted navigation takes this a step further, allowing greater stability compared to freehand navigation due to the rigid robotic arm [9–11]. The benefits of navigated robotic-assisted spine surgery include pre-operative and intra-operative planning, automated trajectory alignment, and less invasive surgery for the patient [8, 10, 12]. Huntsman et al. found an overall successful pedicle screw placement rate of 99% [11].

The current literature is not consistent on whether surgical education has an effect on pedicle screw placement and/or accuracy. Baird et al. compared the surgical skills of a PGY-2, PGY-4 orthopedics resident, a spine surgery fellow, and a board-certified attending spine surgeon [13]. It was concluded that surgeons of differing training levels can safely and accurately place pedicle screws percutaneously. However this was a cadaveric study and only analyzed facet violation and pedicle screw breaches. In comparison, the current study measured planned versus actual pedicle screw placement in a clinical setting.

The arrival of advanced minimally invasive imaging technology may allow residents and fellows to place pedicle screws as accurately as experienced surgeons. In a retrospective radiological study by Laudato et al., 569 thoracolumbar pedicle screws were inserted in 84 patients using three different insertion methods: robotic assistance, O-arm navigation, and freehand lateral fluoroscopy. Screw position was evaluated using Rampersaud criteria, similar to the Gertzbein-Robbins scale used in our study [4, 14]. It was found that supervised spinal fellows inserted pedicle screws with the same accuracy as an experienced spinal surgeon using either O-arm navigation or the freehand technique. Specific planned versus actual offset trajectories were not measured using the robotic assistance technique, so comparison between the senior and junior surgeon was not performed.

### Study limitations

Although this is a retrospective study, the results are consistent with findings from the literature. The number of pedicle screws inserted by residents and attending prior to using the robot is not quantified. Also, pedicle screw accuracy prior to using the robot is unknown. This study forms the foundation for future studies with a higher level of evidence. Comparative studies with larger sample sizes are needed.

## Conclusion

This study demonstrated a high level of accuracy (based on GRS) with no significant differences between the left- and right-side pedicle screw placements (98.67% vs. 97.67%, respectively) in the clinical use of navigated, robot-assisted surgery. In this cohort, both resident and attending surgeon placed pedicle screws successfully under navigated robot guidance.
